# Effects of Grammatical Structure of Compound Words on Word Recognition in Chinese

**DOI:** 10.3389/fpsyg.2018.00258

**Published:** 2018-03-09

**Authors:** Lei Cui, Fengjiao Cong, Jue Wang, Wenxin Zhang, Yuwei Zheng, Jukka Hyönä

**Affiliations:** ^1^Department of Psychology, Shandong Normal University, Jinan, China; ^2^Department of Education and Psychology, Jinan University, Jinan, China; ^3^Department of Psychology, University of Turku, Turku, Finland; ^4^Department of General, Slavic-Russian Linguistics and Classical Philology, Tomsk State University, Tomsk, Russia

**Keywords:** morphological structure, Chinese compounds, subordinate compounds, coordinative compounds, grammatical structure

## Abstract

Two lexical priming experiments were conducted to examine effects of grammatical structure of Chinese two-constituent compounds on their recognition. The target compound words conformed to two types of grammatical structure: subordinate and coordinative compounds. Subordinate compounds follow a structure where the first constituent modifies the second constituent (e.g., 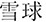, meaning *snowball)*; here the meaning of the second constituent (head) is modified by the first constituent (modifier*)*. On the other hand, in coordinative compounds both constituents contribute equally to the word meaning (e.g., 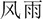, *wind and rain*, meaning *storm* where the two constituent equally contribute to the word meaning). In Experiment 1 that was a replication attempt of Liu and McBride-Chang (2010), possible priming effects of word structure and semantic relatedness were examined. In lexical decision latencies only a semantic priming effect was observed. In Experiment 2, compound word structure and individual constituents were primed by the prime and target sharing either the first or second constituent. A structure priming effect was obtained in lexical decision times for subordinate compounds when the prime and target compound shared the same constituent. This suggests that a compound word constituent (either the modifier or the head) has to be simultaneously active with the structure information in order for the structure information to exert an effect on compound word recognition in Chinese. For the coordinative compounds the structure priming effect was non-significant. When the meaning of the whole word was primed (Experiment 1), no structure effect was observable. The pattern of results suggests that effects of structure priming are constituent-specific and no general structure priming was observable.

## Introduction

In order to understand word recognition in Chinese, a key issue to be resolved is how compound words are identified. Compounding is highly common in Chinese; in fact, the majority of words (72%) are two-character compound words consisting of two free morphemes (Lexicon of Common Words in Contemporary Chinese, 2008). Of all compound words, the vast majority (approximately 93%) are two-constituent compound words (Zhu, 2005)—the kind studied in the present study.

In many Western languages (e.g., English, German, Finnish), the grammatical structure of two-constituent compound words typically conforms to a modifier-head structure: in *snowball* the meaning of the head noun (*ball*) is modified by the constituent preceding the head (*snow*).The way the head is modified by the modifier constitutes the thematic relation between the two constituents. In the case of *snowball*, a MADE OF relation exists between the constituents, that is *ball is made of snow*. On the other hand, a FOR relation exists for *snowtire* (i.e., tire used for snow).

Researchers have assumed the thematic relation information to play an important role in the processing of English compound words (e.g., Kay and Zimmer, 1976; Levi, 1978). More recently, Gagné and Spalding (2004) put forth the CARIN (Competition-Among-Relations-In-Nominals) theory to explain how conceptual combination of compound word constituents contributes to understanding novel noun phrases and compounds. The theory assumes that combined concepts are formed by binding two constituents with a specific thematic relation; the difficulty of any particular combination is a function of the likelihood of the thematic relation for the particular constituents. Moreover, the theory assumes that modifiers are essential to the accessibility of relational information. Relation priming is obtained only when the prime and target shared the same or similar modifier (Gagné, 2001, 2002).

Although the CARIN theory was particularly designed to account for the comprehension of novel compounds, it is meant to be extended to the identification of existing compounds (Gagné and Spalding, 2004). In fact, Spalding and Gagné (2011) showed that compounds such as *snowball* (*ball* MADE OF *snow*) are processed more quickly following a prime that contains the same thematic relation with the target compound (such as *snowfort*; *fort* MADE OF *snow*) than following a prime that conforms to a different thematic relation (such as *snowshovel*; *shovel* FOR *snow*). In order to understand the meaning of compound words, the component meanings need to be brought in contact with each other. The thematic relation of compound words plays a role in that process. In other words, to understand the meaning of *snowball*, the reader needs to understand that it refers to a ball made of snow, whereas *snow tire* is not a tire made of snow but a tire used in snowy conditions. Shoben (1991) has identified 14 different thematic relations between compound word constituents in English.

### The compound word structure in Chinese

All these thematic relations describe the relationship in compound words conforming to the modifier-head structure that is predominant in English (e.g., Katamba, 1993). In Chinese, however, the two constituents form more variable relationships with each other. With respect to their grammatical structure, two-constituent Chinese compounds are divided into five groups: subordinate (e.g., 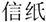, *letter* and *paper*, meaning *notepaper*), coordinative (e.g., 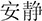, *quiet* and *quiet*, meaning *quiet*), verb-object (e.g., 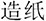, *make* and *paper*, meaning *paper making*), subject-predicate (e.g., 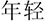, *age* and *light*, meaning *young*), and supplement (e.g., 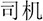, *manage* and *machine*, meaning *driver*) compound words. Subordinate compounds correspond in their structure to the modifier-head structure common in compound words of many Western languages. However, most the other structures are particularly typical of Chinese.

Conceptually, one must keep separate the grammatical structure of Chinese compound words from the thematic relations illustrating how the first constituent modifies the second constituent in modifier-head compounds, as illustrated above. The compound word structure defines the grammatical relation between the components, but it says nothing about their thematic relation. Yet, the grammatical structure is the foundation for establishing a thematic relation between the two constituents (Wang, 2014). In other words, the identification of the grammatical structure must precede that of the more nuanced thematic relation. In languages where the subordinate structure is practically the only structure, only effects of thematic relations can be examined. In the present study, we focused on effects of grammatical structure—a central feature of Chinese compound words. Yet, below we also review studies examining the role of thematic relations in identifying English and Chinese compound words. We do this, because it is not known whether or not the grammatical and thematic relations similarly influence compound word recognition.

Does compound word structure play a role in compound word identification in Chinese? More precisely, is compound word structure utilized during the recognition process? The present study was designed to seek answers to these questions. We examined the recognition of subordinate and coordinative compounds. We focused on these types of compounds, because they are the most common types in Chinese (Yuan and Huang, 1998).

Subordinate compounds are analogous to English or German compounds in that the first constituent is a modifier and the second constituent is the head. On the other hand, coordinative compounds comprise two constituents which contribute equally to the meaning of the whole compound word, neither one modifying the other. Accordingly, there is no semantic dependency relation between them; the two constituents have the same or opposite meaning, and both of them have equal importance to the compound meaning (e.g., 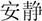, *quiet* and *quiet*, meaning *quiet*). By comparison, subordinate compounds are composed of a modifier and a head, where the modifier is dependent on the head (Manouilidou et al., 2012). All in all, the grammatical relationship between the two constituents is clearly distinct between the two types of compounds.

### The possible role of structural or relational information in compound word processing

The existing theories of compound word recognition differ in whether or not they assign a significant role to the process of conceptually combining the constituents into a unified whole. According to the dual-route theory (e.g., Schreuder and Baayen, 1995; Pollatsek et al., 2000), compound words may be recognized via two parallel routes, the holistic and the decomposition route, that compete with each other. By adopting the holistic route, a compound word is accessed by retrieving its representation in the mental lexicon. Thus, there is no need to activate the individual constituents or a relation between them (Zwitserlood, 1994; Libben, 1998). On the other hand, by adopting the decomposition route compounds are processed in a manner that is similar to the process of combining the constituents into a unified whole (Gagné and Spalding, 2004). Particularly here the structural or relational information may play a role during recognition. It is also possible that for frequent compound words the reader is highly familiar with the whole word meaning, structural or relational information may not play a significant role in the identification process. It may be the case that access to the constituents or the whole-word representation readily yields the compound word meaning without the need for activating or computing the relation between the components (e.g., Libben, 1998). On the other hand, in order to genuinely understand the compound word meaning, the relation between the components needs to be activated or constructed. As pointed out by Libben (2006), the lexical processing system is designed to maximize meaning activation. Undoubtedly, activating (or constructing) the relation between constituents can help readers to realize this goal. Whether or not this is part and parcel of the recognition process is examined in the present study. Before introducing the present study in more detail, we first review key findings obtained for structural and relational effects in compound word recognition.

### Processing of thematic relations in English

Gagné (2002) conducted two priming studies in English to test whether the thematic relation between the constituents in the prime and target compounds affects the processing of modifier-head compounds. For example, the target word *mountain stream* (refers to *stream* located in a *mountain*) was primed either by a compound with an analogous (*mountain cloud* refers to *cloud* located in a *mountain*) or a dissimilar (*mountain magazine* refers to *magazine* about *mountain*) relation between the constituents. The prime and target compounds shared either the same modifier or the same head. She found a constituent repetition effect (a lexical effect) of similar size regardless of whether the prime and the target shared the same modifier or head. More importantly, a relation repetition effect only occurred when the prime and target shared the same relation and the same modifier, but not when they shared the same relation and the same head. This effect was observed for both high-frequent and low-frequent compounds (Gagné and Spalding, 2004, 2006).

Gagné et al. (2009) conducted another priming study to probe whether a relation effect is observed when the shared constituent changed its position between the prime and target. The shared constituent moved from the modifier role in the prime to the head noun role in the target, or vice versa. They found a relation effect only when the shared constituent remained in the same position (and role) across the prime and target.

It appears that in English a relation effect is only established when the prime and target share both the same relation and the same modifier. It is possible that the results may be limited to English and other languages where compounds typically follow the modifier-head structure: the modifier always appears as the first constituent followed by the head. The results may be explained by assuming that the decomposition route is default route in compound word identification. If so, the modifier becomes active prior to its head, inducing an important role for the modifier. What is even more important, the frequency of the modifier's usage of various relations with the head determines the availability of relational information.

### Processing of thematic relations in Chinese

As for Chinese, Ji and Gagné (2007) conducted a sense-nonsense judgment task combined with a priming procedure to probe whether relation information is activated during the processing of Chinese subordinate compounds. Ten different types of thematic relations between modifiers and heads were used in their materials. In Experiment 1, they found a constituent repetition effect and relation priming effect for both the same modifier and the same head conditions. In Experiment 2 and 3, either the modifier or the head was presented 350 ms prior to the other constituent to determine the extent to which relational information is associated with the two constituents. When the modifier preceded the head by 350 ms, relational information associated with the head was still activated, which lends support for the view that in Chinese relational information is not only associated with the modifier. The results for the experiment where the presentation of the head was prolonged were less clear-cut but generally supported the above conclusions regarding the availability of relational information during processing of subordinate compounds.

In a follow-up study, Jia et al. (2013) conducted an ERP (event-related potential) study to examine lexical relation priming in processing Chinese subordinate compound words. They found that relational information is activated when both constituents are accessed, which lends further support for the view that the head is important and necessary for relation priming to occur for Chinese subordinate compound words.

### Processing of grammatical structure in Chinese

Studies investigating the effect of grammatical structure on compound word recognition in Chinese are scarce. Liu and McBride-Chang (2010) conducted a priming experiment combined with the lexical decision task to probe whether structure information can affect the processing of subordinate and coordinative compounds. The prime and target compounds shared the same or different grammatical structure and were either related or unrelated in meaning with each other. The prime appeared 200 ms before the appearance of the target compound. Twenty-four Chinese college students from Hong Kong participated in their study. The experiment was repeated so that lexical decisions were made to the target words in the absence of primes (the no-priming condition). Half of the participants conducted the no-priming condition first, while the other half conducted the priming condition first.

Liu and McBride-Chang (2010) found a marginal interaction between semantic priming, structure priming and compound type. The interaction suggests that for coordinative compounds the shared structure information inhibited the semantic priming effect, whereas for subordinate compounds the same structure boosted the semantic priming effect. The pattern of results was interpreted to suggest that for subordinate compounds “the relationship between lexical processing and morphological structure processing is relatively tight, the processing of morphological structure provides a helpful cue for lexical processing” (p. 614), whereas for coordinative compounds “semantic information alone could activate the lexical representation of targets” (p. 614).

It is noteworthy that the key results were based on statistically marginal interactions, perhaps due to a relatively small sample size (*n* = 21) and significant variation in the standard deviations between conditions (standard deviations in some conditions were double the size of some other conditions). Besides, as the authors point out, Hong Kong students may not have as clear knowledge of the morphological structure of compound words as the students in mainland China who are explicitly taught it in middle school. In the present study, we recruited from a university in mainland China a significantly larger sample (*n* = 96) of participants, who had been taught morphological structure knowledge in their prior studies. It is also noteworthy that in the subsequent ERP study (Chung et al., 2010), structural priming was found to facilitate the processing of semantic information in coordinative compounds (subordinate compounds were not included in the study). The effect was found in the P250 component of ERPs using the prime duration of 57 ms.

The Liu and McBride-Chang (2010) study has provided evidence suggesting that the availability of compound structure information may differently affect the processing of subordinate and coordinative compounds. Yet, the key effects were not robust. Moreover, there is discrepancy in the nature of the observed effects for coordinative compounds. Liu and McBride-Chang (2010) found that the same structure in the prime and target inhibited the semantic priming effect, whereas in the study of Chung et al. (2010) shared structure instead facilitated the semantic priming effect. The discrepancy may be due to the different research methods (response latency vs. ERPs) and/or different priming times (200 ms vs. 57 ms). At any rate, we deemed it important to replicate the Liu and McBride-Chang study using a larger sample of participants recruited in mainland China.

### Present study

Experiment 1 was a replication attempt of the Liu and McBride-Chang (2010) study to examine whether compound structure effects are genuine and can differently affect the processing of subordinate and coordinative compounds. Instead of using the standard priming paradigm, we adopted the paradigm of Gagné (2000), where the participants make lexical decision to both primes and targets. From the participant's perspective, in this paradigm all the presented stimuli are targets, also the ones used as primes. This procedure also ensures that the primes are properly processed, including their structural information, provided that it is an integral part of word identification. The other reason for not using the Liu and McBride-Chang version of the priming paradigm was that they observed a negative priming effect. Lexical decision times were longer when preceded by a prime than when not preceded by a prime. It is not clear what caused this unexpected result. One possibility is that the participants were still processing the prime when the target was presented, which may have lengthened the lexical decision times in the prime condition. We aimed to avoid this unexpected result by giving the participants sufficient time to process the primes. Analogously to Liu and McBride-Chang, both the semantic and structural relation between the prime and target were independently manipulated. In this way we were in the position to find out whether the use of structure information is dependent on semantic information.

The second purpose of the present study was to investigate whether access to structural information is modulated by lexical properties of compound words. Gagné et al. (2009) found priming for thematic relation only when the prime and target shared the same modifier when identifying modifier-head compounds in English. This led them to argue that the modifier plays a key role in determining the thematic relation for subordinate compounds. In Experiment 2, we examined whether priming of grammatical structure of Chinese compound words is mediated by shared constituents between primes and targets. Thus, the prime and target compound words shared either the same first or second constituent and had the same or different word structure. Experiment 2 mimicked the study of Ji and Gagné (2007) with the exception that instead of thematic relations we examined the availability and use of structural information in processing subordinate and coordinative compounds.

## Experiment 1: semantic and structure effects in identifying subordinate and coordinative compounds

Liu and McBride-Chang (2010) found that the same structure boosted the semantic priming effect for subordinate compounds, whereas for coordinative compounds the same structure inhibited the semantic priming effect. However, in their follow-up study using ERPs (Chung et al., 2010), they found that the structure of coordinative compounds instead facilitates the processing of semantic information. We attempted to replicate the Liu and McBride-Chang (2010) study in the hope of being able to shed more light on the discrepant results. We adopted their stimulus materials, but a variant of the priming paradigm where lexical decisions were made both to prime and target compound words.

### Methods

#### Participants

One hundred-five undergraduate students from Shandong Normal University participated in the study. They were all native speakers of Chinese with normal or corrected-to-normal vision.

#### Materials and design

We adopted the 61 word prime—word target stimuli (32 subordinate compound words, 29 coordinative compound words) and 60 word prime—non-word target pairs used by Liu and McBride-Chang (2010). For pretesting the stimuli, the reader should consult the original study for further details. The design was a 2 (Compound Structure: subordinate and coordinative) × 2 (Semantic Priming Condition: semantically related vs. semantically unrelated) × 2 (Structure Priming Condition: same structure vs. different structure) within-participants design.

For the subordinate target compound words, the condition with a similar meaning and same structure (SMSS) refers to one where the target (e.g., 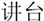, *speak* and *platform*, meaning *platform*) and the prime (e.g., 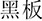, *black* and *board*, meaning *blackboard*) are related in meaning and comprise the same subordinate structure. The condition with a similar meaning and different structure (SMDS) refers to one where the target (e.g., 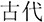, *ancient* and *generation*, meaning *ancient*) and the prime (e.g., 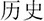, *calendar* and *history*, meaning *history*) are related in meaning, but their compound structures differ (e.g., the prime has a coordinative structure). The condition with a different meaning and same structure (DMSS) refers to one where the target (e.g., 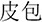, *fur* and *bag*, meaning *wallet*) and the prime (e.g., 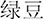, *green* and *bean*, meaning *mung bean*) are unrelated in meaning, but have the same subordinate structure. Finally, the condition with a different meaning and different structure (DMDS) refers to one where the target (e.g., 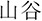, *mountain* and *gorge*, meaning *valley*) and the prime(e.g., 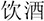, *drink* and *liquor*, meaning *drink*) are neither related in meaning nor have similar compound structure (e.g., the prime has a verb-object structure).

Analogous conditions were created for the coordinative target compound words. The condition with a similar meaning and same structure (SMSS) refers to one where the target (e.g., 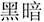, *black* and *dim*, meaning *darkness*) and the prime (e.g., 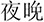, *night* and *late*, meaning *night*) are related in meaning and comprise the same coordinative structure. The condition with a similar meaning and different structure (SMDS) refers to one where the target (e.g., 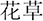, *flower* and *grass*, meaning *plants*) and the prime (e.g., 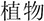, *plant* and *thing*, meaning *plant*) are related in meaning, but their compound structures differ (e.g., the prime has a subordinate structure). The condition with a different meaning and same structure (DMSS) refers to one where the target (e.g., 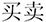, *buy* and *sell*, meaning *deal*) and the prime (e.g., 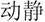, *move* and *still*, meaning *sound*) are unrelated in meaning, but have the same coordinative structure. Finally, the condition with a different meaning and different structure (DMDS)refers to one where the target (e.g., 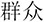, *group* and *flock*, meaning *crowd*) and the prime (e.g., 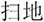, *sweep* and *floor*, meaning *floor sweeping*) are neither related in meaning nor have similar compound structure (e.g., the prime has a verb-object structure).

On a 10-point scale, the average rating of the semantically related conditions was around 7 and that of the unrelated conditions around 1. The target words were chosen from among the top 6,000 words (roughly corresponding to the written language vocabulary size of an average middle-level Chinese speaker) from the database of Da (2004). There were no significant differences in frequency for the targets or primes between the four priming conditions. For the subordinate compound words, the target frequencies are 0.06097, 0.12494, 0.06168, and 0.14212 for the SMSS, SMDS, DMSS, DMDS conditions, respectively (see Table 1 of Liu and McBride-Chang). The corresponding prime frequencies are 0.07004, 0.14212, 0.06936 and 0.17685. For the coordinative compound words, the target frequencies are 0.14808, 0.20454, 0.11922, and 0.07384 for the SMSS, SMDS, DMSS, DMDS conditions, respectively (see Table 1 of Liu and McBride-Chang). The corresponding prime frequencies are 0.11922, 0.07384, 0.05218, and 0.07288. In addition, Liu and McBride-Chang asked 10 college students to select the structure of the words from several alternatives. The selection accuracies were high for both the subordinate (81.42%) and coordinative (90.41%) compound words, even though their difference was significant. The structure of the subordinate compound words was more difficult to identify than that of coordinative compound words. Another set of 45 students were asked to access the semantic transparency of experimental materials. The rating revealed no difference between the subordinate and coordinative compound words.

Apart from these materials, 60 non-word prime—word target pairs and 60 non-word prime—non-word target pairs were also generated to equate the number of words and non-words in the experimental materials. The non-word stimuli were created from real coordinative compounds by replacing one character from a coordinative compound with another one from another coordinative compound. An analogous procedure was used with subordinate compounds. With the change of one character of the two-character real words, the new combination of the two characters did not have a clear or common meaning in everyday spoken Chinese. For example, the nonword 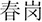 (*spring* and *duty*) was formed by changing the second character of the real word 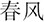 (*spring* and *wind*, meaning *spring breeze*). Thus, by being non-meaningful the grammatical structure of the nonwords could not be readily discerned.

Apart from the materials used in the experiment proper, 8 pairs of other items were used as practice items. Moreover, 12 pairs of fillers were used at the beginning of each experimental block. In all prime-target pairs, no constituent morpheme was shared across the pairs.

#### Apparatus and procedure

E-Prime 2.0 software was used to program the experiment and record participants' reaction times and errors. All materials were presented in 38-point size simple Song font at the center of the computer screen in white on a black background. Each character was about 2.1 × 2.1 cm^2^ in size. The viewing distance of the participant to the screen was 60 cm. At this distance, each character subtended approximately 2° of visual angle.

We adopted the same experimental procedure as Gagné (2000). Participants performed a lexical decision task both with primes and targets. Trial presentation was self-paced. Participants were asked to judge whether the stimulus was a word or a nonword as quickly and accurately as possible. Participants sat in front of a computer screen and placed the index finger of their left hand on the F key of the computer keyboard and the index finger of their right hand on the J key. The keys were labeled. For half of the participants, the F key corresponded to Word and the J key corresponded to Nonword. The reverse was true for the other half of the participants. As in the Liu and McBride-Chang (2010) study, in order to control for the variance due to the nature of the different items, participants were asked to carry out the experiment with and without primes. Half of the participants completed the no-prime experiment first, and the other half carried out the priming experiment first.

In the priming experiment, each trial began with the message “ 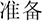
” (*ready* in English) presented on the computer screen. The participant pressed the space bar to display the item in the center of the screen. First, the prime item appeared and participants indicated whether or not the compound had a sensible interpretation (a real word) by pressing the appropriate key. After the participant had responded, the target compound was displayed in the same manner. There was nothing in the manner of display to indicate which items were primes and which items were targets or that the prime and target compounds were related. In the no-prime experiment, the procedure was the same as in the priming experiment, with the only difference being that the primes were excluded. There was a 2-min break between the priming and non-priming trials, and also in the middle of the two priming conditions. The experiment took about 45 min to complete.

## Results

Nine participants were excluded because their comprehension accuracy was below 80%. For the remaining 96 participants included in the analyses, the mean comprehension accuracy in the priming block was 92.4% and in the no-priming block 93.6%. Reaction times more than 3 standard deviations were removed (1.95% of correct responses). Following Liu and McBride-Chang (2010), a difference score was computed for each target compound by subtracting the reaction time obtained in the priming block for the word from that obtained in the no-priming block. Analyses of variance (ANOVAs) were conducted on these difference scores. ANOVAs were computed by participants (*F*_1_) and by items (*F*_2_). Descriptive statistics for the different stimulus condition are shown in Table 1. As is evident from Table 1, response accuracy approached ceiling in all conditions. There were no significant main effects or interactions for response accuracy, *Fs* < 2.04.

**Table 1:** Response accuracy (%) and mean reaction time (in ms) for the no-prime and prime block and their difference (D).

**Word type**	**Priming condition**	**Accuracy**			**Reaction time**		
		**No-prime**	**Prime**	**D**	**No-prime**	**Prime**	**D**
Subordinate	SMSS	96.13 (0.04)	98.21 (0.02)	−2.08	739 (214)	696 (206)	42
	SMDS	96.61 (0.03)	99.96 (0.01)	−3.35	738 (224)	679 (191)	60
	DMSS	96.88 (0.03)	98.18 (0.02)	−1.30	703 (202)	680 (180)	23
	DMDS	97.69 (0.02)	98.15 (0.02)	−0.46	693 (179)	675 (179)	18
Coordinative	SMSS	98.15 (0.02)	98.84 (0.01)	−0.69	694 (197)	652 (173)	42
	SMDS	98.07 (0.02)	98.66 (0.01)	−0.59	696 (194)	648 (163)	47
	DMSS	98.96 (0.01)	99.31 (0.01)	−0.35	689(180)	673 (195)	16
	DMDS	97.92 (0.02)	98.36 (0.02)	−0.44	720 (207)	681 (183)	39

*Standard deviations in parenthesis. SMSS, similar meanings, same structure; SMDS, similar meanings, different structure; DMSS, dissimilar meanings, same structure; DMDS, dissimilar meanings, different structure. Differences (D) were calculated as the difference scores of no-prime compared to each priming condition*.

**Figure 1:**
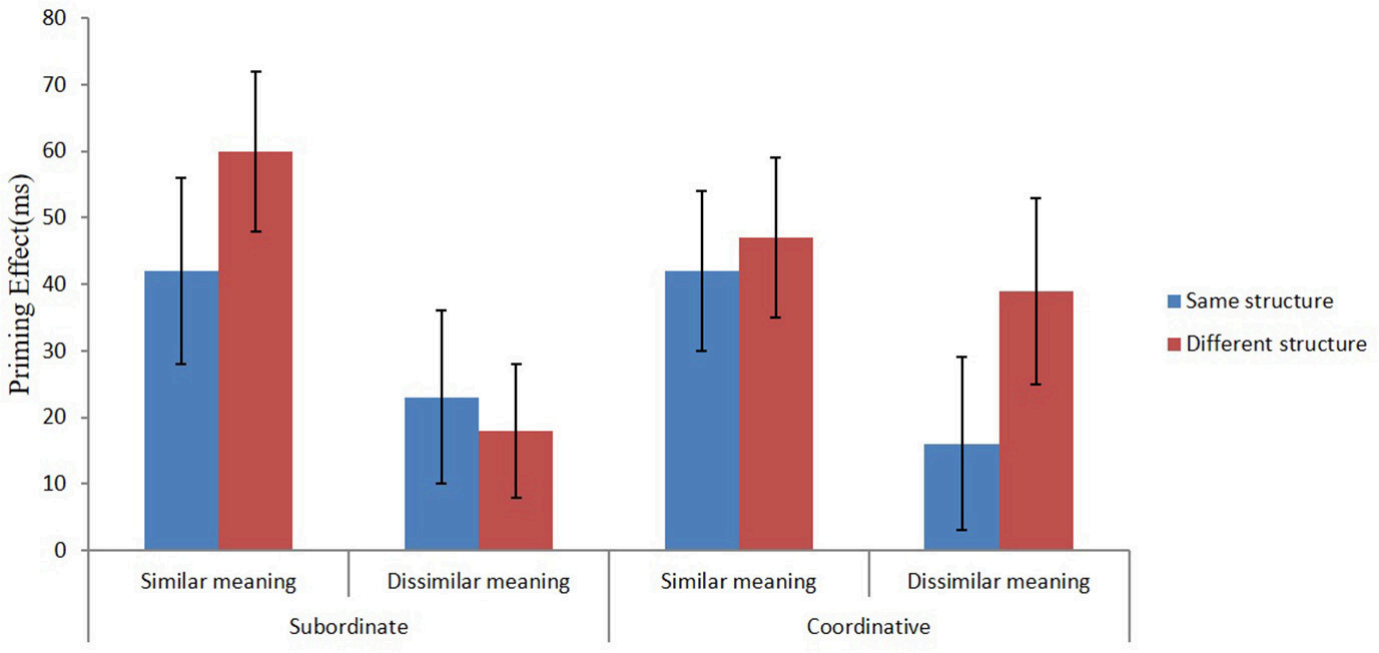
The size of the priming effect (ms) for the subordinate and coordinative compounds, calculated as the time differences of the no-prime compared to the corresponding prime conditions (no-prime–prime) in Experiment 1,when the prime and target had the same or different structure and when the prime and target shared either similar or dissimilar meaning.

In reaction times (see Table 1), there was a significant main effect of the semantic priming condition, *F*_1(1, 95)_ = 12.79, *MS*_*e*_ = 11,220, *p* < 0.01; *F*_2(1, 53)_ = 12.08, *MS*_*e*_ = 49,317, *p* < 0.01. The priming effect (the difference between the no-prime and the prime block) was larger for the semantically related priming condition than for the semantically unrelated condition (a difference of 94 ms; see Figure 1 for the graphic depiction of the size of the priming effect). There was no significant main effects of compound structure or the structure priming condition, or two-way or three-way interactions, *Fs* < 1. Following Liu and McBride-Chang (2010), we nevertheless conducted separate ANOVAs for subordinate and coordinative compounds.

For subordinate compounds, there was a significant main effect of the semantic priming condition, *F*_1(1, 95)_ = 11.25, *MS*_*e*_ = 8,318, *p* < 0.01; *F*_2(1, 28)_ = 7.74, *MS*_*e*_ = 35,072, *p* < 0.01. The priming effect was larger for the semantically related priming condition than for the semantically unrelated condition (a difference of 60 ms; see Figure 1). All other effects were non-significant, *F*s < 1.

For coordinative compounds, there was a significant main effect of the semantic priming condition, *F*_1(1, 95)_ = 4.44, *MS*_*e*_ = 11,876, *p* < 0.05; *F*_2(1, 25)_ = 6.53, *MS*_*e*_ = 12,994, *p* < 0.05. The priming effect was larger for the semantically related priming condition than for the semantically unrelated condition (difference = 34 ms). Other effects were non-significant, *F*s < 1.91.

## Discussion

Experiment 1 was a direct replication of the methodology of Liu and McBride-Chang (2010) study with the exception that instead of using the standard priming paradigm, we adopted the procedure of Gagné (2000), where lexical decision was made both to primes and targets. We reasoned that it would boost the activation of structure information during the priming phase and lead to more robust structure effects.

The main result of Experiment 1 is that we failed to find structural priming for either subordinate or coordinative Chinese compound words. The only reliable effect was a semantic priming effect. Recognition of target compound words was faster when preceded by a semantically related compound prime than when the prime stimulus was absent. The structure effect was absent both when the prime and target were semantically related or unrelated. This is in contrast to Liu and McBride-Chang (2010), who found structure priming to interact with semantic priming for subordinate compounds.

As identical stimulus materials were used across the two studies, the discrepancy in the results cannot reflect the choice of stimuli. Instead, it is more likely to reflect differences in sample size. To secure sufficient statistical power to find a structure effect, we tested nearly 100 participants, whereas the sample of Liu and McBride-Chang was limited to 21 participants. We think it is unlikely that the lack of finding a structure effect would be due to insufficient power in the present study. It is also possible that the discrepancy may reflect differences in the experimental paradigms used. However, it should be noted that we employed a paradigm that has been shown to be sensitive to relation effects (Gagné and Spalding, 2004, 2009; Ji and Gagné, 2007; Jia et al., 2013). Thus, it is unlikely that our experimental procedure would have been insensitive to observe structure effects. Whatever the ultimate reason may be for the discrepant results, it seems genuine structure effects in Chinese compound word recognition may either be non-existent or hard to get.

Prior studies establishing a thematic priming effect in compound word recognition (Gagné and Spalding, 2004, 2009; Ji and Gagné, 2007; Jia et al., 2013) have demonstrated that relation priming occurs when the prime and the target share a same constituent. These results may be taken to suggest that the activation of relation information is constituent-specific; in other words, abstract relation priming would not exist. Thus, Experiment 2 was designed to examine whether this is true also for priming of grammatical structure of Chinese compound words.

## Experiment 2: constituent repetition and structure effects for coordinative and subordinate compounds

The purpose of Experiment 2 was to investigate whether an effect of compound word structure can be established when the prime and the target compounds share the same first or second constituent in coordinative and subordinate compounds. In Experiment 2A this was done with coordinative compounds and in Experiment 2B with subordinate compounds.

In studies concerning the availability of relational information during processing English modifier-head (i.e., subordinate) compounds, relational information associated with the modifier has been influential. On the other hand, the study conducted by Ji and Gagné (2007) with Chinese subordinate compounds provided support for the importance of heads. This makes sense, as the grammatical structure of Chinese compound words is variable, so that the reader cannot judge on the basis of the first constituent the type of compound word. Instead, (s)he also needs to access the head. Moreover, Chinese is written with no spaces, so word boundaries are not visually marked. Thus, when the reader encounters a character, it is not immediately clear whether it is a constituent of a compound word or a free-standing morpheme. This in turn leads to the need for constructing compounds online (Myers, 2006).

With respect to subordinate compounds, it is also possible that access to the grammatical structure of subordinate compounds becomes available via the modifier, as was found to be the case for thematic relation in English compound words. If so, a shared modifier between the prime and target should speed up the recognition of Chinese subordinate compounds more than a shared head. On the other hand, if we are to replicate the results of Ji and Gagné (2007) observed for thematic relation in Chinese subordinate compounds, a shared modifier and a shared head between the prime and target should speed up target recognition to a similar degree. The predictions concerning the coordinative compounds are less clear-cut. If we are to replicate the result of Experiment 1, we will not observe structure priming even when the prime and target share a constituent. On the other hand, as both constituents contribute equally to the compound word meaning, it may also be predicted that the priming effect is equally strong when either the first or second constituent is shared between the prime and the target.

## Experiment 2A: constituent repetition and structure effects for coordinative compounds

Gagné (2001) found for English compounds a relation effect only when the prime and the target shared the same modifier. However, Ji and Gagné (2007) showed that in processing Chinese subordinate compounds relational information associated also with the head becomes available. In Experiment 2A, we examined whether an effect of the grammatical structure of Chinese compound words can be established for coordinative compounds when the prime and the target compounds shared the same first or second constituent.

### Methods

#### Participants

Forty-six undergraduate students from Shandong Normal University participated in the study. They were all native speakers of Chinese with normal or corrected-to-normal vision.

#### Materials and design

Eighty coordinative compounds were selected from the Modern Chinese Word Dictionary (2005) as the targets. Five kinds of priming compounds were constructed for each target compound (e.g., 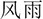, *wind and rain*, meaning *storm*). They varied in terms of whether or not they shared the same constituent and the same structure. In the same structure condition, the prime either shared the same first constituent with the target (SFSS, e.g., 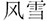, *wind and snow*, meaning *blizzard*), or the same second constituent with the target (SSSS, e.g., 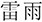, *thunder and rain*, meaning *thunderstorm*); for the different structure condition, the prime either shared the same first constituent with the target (SFDS, e.g., 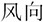,*wind and direction*, meaning *wind direction*), or the same second constituent with the target (SSDS, e.g., 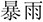, *violent and rain*, meaning *rainstorm*). There was also a neutral priming condition as the baseline that neither shared the same structure nor the same constituent with the target (Neutral, e.g., 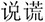, *speak and lie*, meaning *tell a lie*). The grammatical structures of primes conformed either to the subordinate, verb-object, subject-predicate or supplement structure. The first character of each target compound was matched on the number of strokes and frequency. The five priming compounds were matched on the word frequency, the number of strokes and frequency of the first character, as well as the number of strokes and frequency of the second character. Descriptive statistics for the different stimulus conditions are shown in Table 2.

**Table 2:** Lexical-statistical properties of five kinds of priming compounds for coordinative compounds (Experiment 2A).

**Priming condition**	**Word frequency**	**Frequency of first character**	**Frequency of second character**	**Number of strokes in first character**	**Number of strokes in second character**	**Semantic relation (%)**
SFSS	10.76 (27)	222(638)	149 (437)	8.70 (3.0)	8.88 (2.9)	60 (1.01)
SFDS	7.73 (44)	222 (638)	278 (791)	8.70 (3.0)	8.30 (2.7)	48 (0.95)
SSSS	7.48 (21)	406 (1330)	270 (1132)	8.50 (3.0)	8.15 (2.3)	60 (0.84)
SSDS	4.19 (12)	687 (2782)	270 (1132)	8.01 (2.9)	8.15 (2.3)	48 (0.83)
Neutral	2.28 (5)	291 (1434)	132 (275)	8.20 (2.6)	8.76 (2.8)	28 (0.33)

*Standard deviations are presented in parentheses. Word frequency and character frequency are given as a characters-per-million figure (Cai and Brysbaert, 2010). Semantic relation between the prime and target is given as a percentage. SFSS, same first constituent, same structure; SFDS, same first constituent, different structure; SSSS, same second constituent, same structure; SSDS, same second constituent, different structure; Neutral, different constituent, different structure*.

Five lists were constructed. Each list contained an equal number of items from each priming condition. The items were counterbalanced using a Latin square design such that the participants saw each target compound only once.

Matching the compound word structure and the constituents between the prime and the target word heavily constrained what could be selected as the priming compounds. Despite this, efforts were made to minimize differences in semantic relationship between the priming conditions. We investigated the semantically relationship between the target compound and the five primes. Ninety participants who did not take part in the experiment rated the semantic relationship using a 5-point scale (1 = Unrelated; 5 = Related). Semantic relation between the primes and targets is given as a percentage figure in Table 2. Twenty filler character pairs with a similar format and a strong semantic relation were also included. Five lists of 100 compound pairs were constructed. Each list contained an equal number of items from each condition. The items were counterbalanced using a Latin square design such that the participants saw each compound pair only once. Participants were randomly allocated to each list.

Despite the efforts to minimize differences in the semantic relationship we nevertheless found a significant main effect of priming condition, *F*_(4, 395)_ = 85.80, *MS*_*e*_ = 32.52, *p* < 0.05. Post hoc tests showed that semantic relationship between the prime and target compounds was most distant for the neutral condition (*t*s > 9.37, *p*s < 0.05). In the two conditions with the same structure (SSSS and SFSS), primes and targets were semantically closer to each other than in the different structure (SSDS and SFDS) conditions (*t*s > 4.63, *p*s < 0.05). However, there was no difference between the SSSS and SFSS conditions, *t*_(79)_ = 0.10, *p* > 0.1, or the SSDS and SFDS conditions, *t*_(79)_ = 0.17, *p* > 0.1.

Apart from the experimental materials, 80 non-word prime—word target pairs, 80 word prime—non-word target pairs and 80 non-word prime—non-word target pairs were also generated to equate the number of words and non-words in the stimulus set. The generation of non-words was done as in Experiment 1. Moreover, 8 pairs of items were used as practice items and 12 pairs of fillers were used at the beginning of each experimental block.

#### Apparatus and procedure

The same apparatus was used as in Experiment 1. The procedure was analogous to the one used in Experiment 1. The experiment took about 35 min to complete.

### Results

Six subjects were excluded because their comprehension accuracy was below 70%. For the remaining 40 subjects included in the analyses, the mean comprehension accuracy was 92.6%. Reaction times more than 2.5 standard deviations were removed (2.63% of correct responses). Descriptive statistics for the different experimental conditions are shown in Table 3.

**Table 3:** Accuracy (%) and mean reaction time (ms) for Experiment 2.

**Word type**	**Priming condition**	**Accuracy**	**Reaction time**
Coordinative compounds	SFSS	98 (0.03)	721 (100)
	SFDS	98 (0.04)	735 (117)
	SSSS	98 (0.04)	729 (86)
	SSDS	97 (0.05)	732 (105)
	Neutral	96 (0.08)	798 (126)
Subordinate compounds	SFSS	99 (0.04)	657 (96)
	SFDS	98 (0.04)	671 (90)
	SSSS	98 (0.04)	663 (92)
	SSDS	98 (0.03)	683 (102)
	Neutral	96 (0.06)	740 (97)

*Standard deviations are in parentheses*.

*SFSS, same first constituent, same structure; SFDS, same first constituent, different structure; SSSS, same second constituent, same structure; SSDS, same second constituent, different structure; Neutral, different constituent, different structure*.

In order to see whether the priming condition differed from the neutral condition, we first ran a one-way ANOVA with 5 priming conditions. Response accuracy was at ceiling, and thus there were no differences between 5 priming conditions, *F*_1(4, 195)_ = 0.943, *MS*_*e*_ = 0.019, *p* = 0.440; *F*_2(4, 396)_ = 0.966, *MS*_*e*_ = 0.009, *p* = 0.426. In the reaction times, there was a significant main effect of the priming condition, *F*_1(4, 195)_ = 10.83, *MS*_*e*_ = 3,629, *p* < 0.001; *F*_2(4, 396)_ = 9.24, *MS*_*e*_ = 14.49, *p* < 0.001. Reaction times were longer for the neutral condition than the other four conditions (*ts* > 4.14, *ps* < 0.001).

To probe whether structure priming occurred and whether constituent repetition interacted with structure priming, we analyzed the data using a 2 (constituent repetition: first vs. second) × 2 (structure: same vs. different) design, in other words, excluding the neutral prime condition. No significant main effects or interactions were found either for response accuracy or reaction time (*F*s < 1.58). In other words, for coordinative compound there was no structure effect regardless of which constituent was repeated across the prime and target.

## Experiment 2B: constituent repetition and structure effects for subordinate compounds

Experiment 2B was analogous to Experiment 2A except that subordinate compounds were used as stimuli. The participant sample was also different from that of Experiment 2A. Ji and Gagné (2007) established a relational effect for subordinate compounds when both the modifier and the head were shared across the prime and target. Experiment 2B examined whether this is also the case for structure information.

### Methods

#### Participants

Fifty-eight undergraduate students from Shandong Normal University participated in the study. They were all native speakers of Chinese with normal or corrected-to-normal vision.

The column headings were missing from Table 4. The column headings are (from left to right): Priming condition; Word frequency; Frequency of first character; Frequency of second character; Number of strokes in first character; Number of strokes in second character; Semantic relation (%) The order of lines in Table 4 should also be changed (from top to bottom): SFSS, SFDS, SSSS. SSDS.

**Table 4:** Lexical-statistical properties of five kinds of priming compounds for subordinate compounds (Experiment 2B).

**Priming condition**	**Word frequency**	**Frequency of first character**	**Frequency of second character**	**Number of strokes in first character**	**Number of strokes in second character**	**Semantic relation (%)**
SFSS	2.93 (4.43)	444 (1707)	266 (977)	8.57 (3)	8.39 (3.28)	62 (1.07)
SFDS	4.62 (9.88)	444 (1707)	1045 (606)	8.57 (3)	7.8 (2.85)	46 (0.96)
SSSS	2.84 (4.76)	154 (282)	506 (1430)	8.61 (3.57)	8.31 (2.87)	57 (0.77)
SSDS	6.85 (16.66)	312 (1063)	506 (1430)	8.19 (3)	8.31 (2.87)	43 (0.83)
Neutral	2.95 (5.09)	102 (305)	130 (560)	8.76 (2.68)	8.93 (3.66)	27 (0.32)

*Standard deviations in parentheses. Word frequency and character frequency is measured as a characters-per-million figure (Cai and Brysbaert, 2010). Semantic relation between the prime and target is given as a percentage. SFSS, same first constituent, same structure; SFDS, same first constituent, different structure; SSSS, same second constituent, same structure; SSDS, same second constituent, different structure; Neutral, different constituent, different structure*.

#### Materials and design

Seventy subordinate compounds were selected from the Modern Chinese Word Dictionary (2005) as the targets. Five priming conditions were constructed for each target compound (e.g., 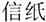, *letter and paper*, meaning *notepaper*). The primes varied in terms of whether the modifier or the head was shared between the prime and the target and whether the compound word structure was the same or different between the prime and the target. When they shared the same head (i.e., the second constituent), the structure was either the same (SSSS; e.g., 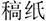, *draft and paper*, meaning *manuscript paper*), or different (SSDS; e.g., 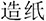, *make and paper*, meaning *paper making*). Analogously, when they shared the same modifier (i.e., the first constituent), the prime either shared the same (SFSS; e.g., 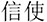, *letter and make*, meaning *messenger*) or different structure (SFDS, e.g., 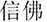, *believe and Buddha*, meaning *Buddhist*) with the target. Moreover, there was also a neutral prime condition as the baseline that neither shared the same structure nor shared the same constituent with the target (Neutral, e.g., 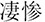, *sad and tragic*, meaning *miserable*). The different structure primes conformed either to the verb-object, subject-predicate, coordinative or supplement structure. The five priming compounds were matched on the frequency of the compound words, the number of strokes and frequency of the first character, as well as the number of strokes and frequency of the second character (*F*s < 1.98). Descriptive statistics for the different stimulus conditions are shown in Table 4.

Five lists were constructed. Each list contained an equal number of items from each priming condition. The items were counterbalanced using a Latin square design such that the participants saw each target compound only once.

Similarly to the coordinative compounds, efforts were made to minimize differences in the semantic relationship between the primes and targets given the constraints related to compound word structure and constituent repetition. Ninety participants rated the semantic relationship between the target and the five primes using a 5-point scale (1 = Unrelated; 5 = Related). Semantic relation is given as a percentage figure in Table 4. Fourteen filler character pairs with a similar format and a strong semantic relation were also included. Five lists of 84 compound pairs were constructed. Each list contained an equal number of items from each condition. The items were counterbalanced using a Latin square design such that the participants saw each compound pair only once. Participants were randomly allocated to each list.

Despite efforts to minimize differences in the semantic relationship between the primes and targets we nevertheless found a significant main effect of priming condition, *F*_(4, 345)_ = 76.51, *MS*_*e*_ = 26.34, *p* < 0.05. Post hoc tests showed that the semantic relationship between the primes and the targets was lowest for the neutral condition than for the other conditions (*t*s > 7.6, *p*s < 0.05). Similarly to the coordinative compounds, also for subordinate compounds the primes and targets sharing the same structure (SSSS and SFSS) were semantically closer to each other than those having a different (SSDS and SFDS) structure (*t*s > 3.81, *p*s < 0.05). However, no differences were observed between the SSSS and SFSS conditions, *t*_(69)_ = 1.64, *p* > 0.10, or the SSDS and SFDS conditions, *t*_(69)_ = 0.93, *p* > 0.10.

Apart from the experimental materials, 70 non-word prime—word target pairs, 70 word prime—non-word target pairs and 70 non-word prime—non-word target pairs were also generated to equate the number of words and non-words in the stimulus set. The generation of non-words was done as in Experiment 1. Moreover, 8 pairs of additional items were used as practice items and 12 pairs of fillers were used at the beginning of each experimental block.

#### Apparatus and procedure

The same apparatus was used as in Experiment 1. The procedure was analogous to the one used in Experiment 1. The experiment took about 30 min to complete.

### Results

Eight subjects were excluded because their comprehension accuracy was below 70%. For the remaining 50 subjects included in the analyses, the mean comprehension accuracy was 91.5%. Reaction times more than 2.5 standard deviations were removed (5.09% of correct responses). Descriptive statistics for the different stimulus condition are shown in Table 3.

We first ran a one-way ANOVA with all 5 priming conditions. For response accuracy, there was a significant main effect of the priming condition, *F*_1(4, 245)_ = 4.030, *MS*_*e*_ = 0.062.002, *p* < 0.01; *F*_2(4, 346)_ = 2.845, *MS*_*e*_ = 0.032, *p* < 0.05. Response accuracy was lower in the neutral condition than the other four conditions (*ts* > 2.12, *ps* < 0.038).For reaction times, there was a significant main effect of the priming condition, *F*_1(4, 245)_ = 20.30, *MS*_*e*_ = 2,728, *p* < 0.001; *F*_2(4, 346)_ = 12.65, *MS*_*e*_ = 13.62, *p* < 0.001. Reaction times were longer in the neutral condition than the other four conditions (*ts* > 5.14, *ps* < 0.001).

Similarly to Experiment 2A, we subsequently computed 2 (constituent repetition: first vs. second) × 2 (structure: same vs. different) ANOVAs. For response accuracy, no significant main effects or interactions were found (*F*s < 1.41).For reaction time, there was a significant main effect of structure, *F*_1(1, 49)_ = 6.27, *MS*_*e*_ = 2,299, *p* < 0.05; *F*_2(1, 69)_ = 6.09, *MS*_*e*_ = 4,064, *p* < 0.05; reaction times were 17 ms shorter in the same structure condition than in the different structure condition. The structure effect was significant for both the same head [an effect size of 20 ms; *t*_1(49)_ = 1.71, *p* < 0.05; *t*_2(69)_ = 2.01, *p* < 0.05] and the same modifier [an effect size of 14 ms; *t*_1(49)_ = 1.55, *p* = 0.06; *t*_2(69)_ = 1.86, *p* < 0.05] conditions. The main effect of constituent repetition type and the two-way interaction remained non-significant (*F*s < 1.56). In other words, it did not make a difference whether the modifier or the head was repeated.

### Discussion of experiment 2A and 2B

In Experiment 2, effects of grammatical structure on compound word recognition in Chinese were studied using a priming paradigm where structure and constituent priming were independently manipulated. In Experiment 2A, this was done using coordinative compounds as the stimuli, while in Experiment 2B the stimuli were subordinate compounds. Both experiments established a lexical-semantic priming effect: by repeating the first or second constituent across the prime compound and the target compound facilitated word recognition in comparison to when the prime comprised different constituents than the target. Our results showed that there is constituent repetition priming regardless of the compound structure and regardless of whether the first or second constituent was repeated. The results are consistent with previous studies (Zwitserlood, 1994; Libben et al., 2003; Ji and Gagné, 2007). This suggests that access to compound word constituents is an integral part of compound word recognition in Chinese. In other words, the decomposition route is active during the recognition process.

**Figure 2:**
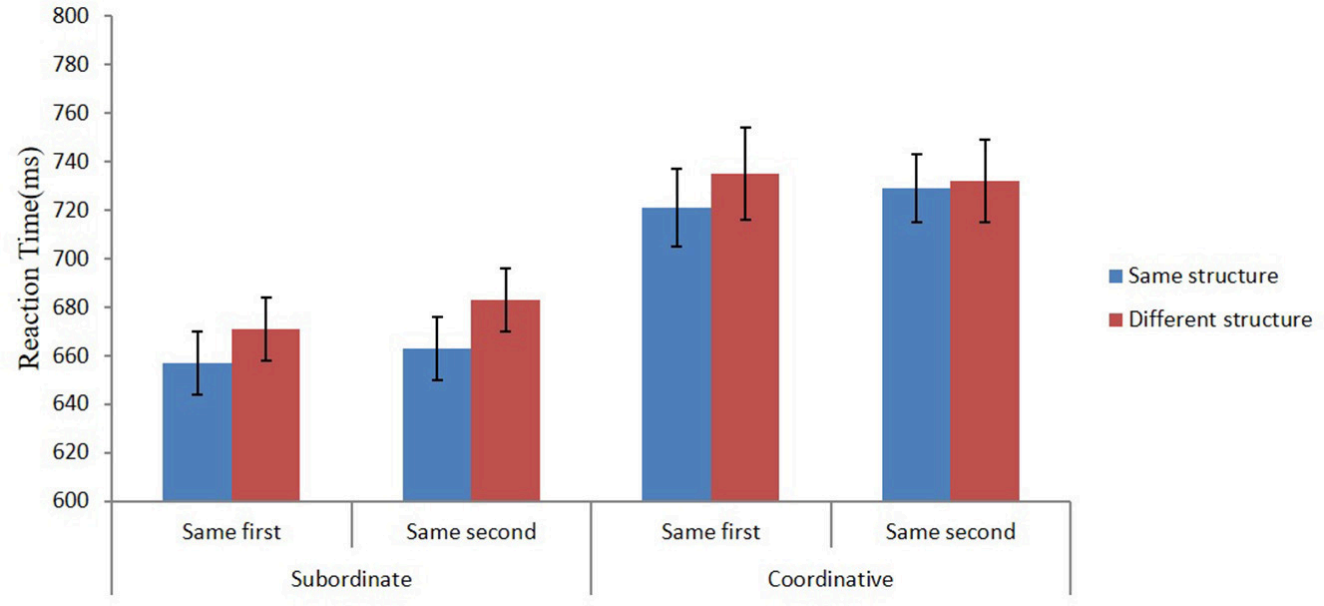
Mean reaction times (ms) for the subordinate and coordinative compounds in Experiment 2 when the prime and target had the same or different structure and when the prime and target shared either the first or second constituent. Error bars represent standard errors of mean.

More importantly for the present study, an effect of grammatical structure was established for subordinate compounds but not for coordinative compounds. The pattern of results for the lexical decision times is illustrated in Figure 2. In subordinate compounds, the structure effect was reliable both when the modifier and the head was repeated. This compares favorably with the results of Ji and Gagné (2007) observed for the utilization of thematic relations during processing of Chinese subordinate compounds. They demonstrated that relational information associated with both the modifier and the head becomes available during word recognition. Similar results were reported by Jia et al. (2013). It is noteworthy that the relational effect they obtained in Experiment 1 that was similar to our Experiment 2B was about twice the size we observed for structural information (32 vs. 17 ms).

Efforts were made to equate the overall semantic relationship between the prime and target compounds. Nevertheless, in both experiments the prime and target were semantically closer to each other when they shared a constituent than when they did not share a constituent. This is understandable, as in the repetition condition an identical constituent appeared both in the prime and target. Perfect matching may in fact be impossible. Thus, the difference between the neutral and the shared constituent conditions is likely to reflect a combination of a lexical repetition and a semantic effect. Ji and Gagné (2007) do not provide any information about the semantic overlap between primes and targets. It is likely that the aforementioned confound applies also to their study. However, we consider it unlikely that the structure priming effect obtained for subordinate compounds should be interpreted as a semantic effect for two reasons. First, an analogous semantic relation existed between the primes and targets for coordinative compounds; yet, no effect was observed. Second, in Experiment 1, the semantic relationship between the prime and target did not modify the structure effect.

In Experiment 2A, we found no structure effect of coordinate compounds. They are compounds that consist of constituents that contribute equally to the meaning of the compound word. The constituent meanings are either similar or opposite to one another and their combination constitutes the word meaning (e.g., 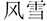, *wind snow*, meaning *blizzard*). Why was no structure effect obtained for coordinative compounds?

Manouilidou et al. (2012) note that in subordinate compounds an apparent and consistent relation exists between the constituents, as the first one always modifies the second. However, in coordinative compounds there is no such dependency relation between the two constituents. Moreover, the relationship between the two constituents is not consistent, as they can be either of similar or opposite meaning to one another. Thus, due to the lack of apparent and consistent relationship between the constituents, no structure priming emerged for coordinative compounds^1^.

It is also possible that the word meaning is activated by the meaning of the individual characters, in which case the relationship between the constituents does not need to be elaborated. This may be particularly the case with coordinative compounds whose constituents have similar meaning. Evidence for this view comes from a study conducted with coordinative Greek compounds by Manouilidou et al. (2012). They found stronger lexical priming for both first and second constituents for coordinative than for subordinate Greek compounds.

It should also be noted that although the effect of grammatical structure was non-significant for coordinative compounds, as is evident from Figure 1, there is a trend for a structure effect particularly when the prime and target shared the first constituent. Thus, there is not a qualitative difference in how structure effects play out for coordinative and subordinate compounds. It is just that the effect is clearer for subordinate compounds.

Finally, in addition to the speculations above regarding possible reasons for not finding a reliable structure effect for coordinative compounds, it is also possible that the effect is constituent-specific. Constituents vary in frequency with which they are involved in different grammatical structures either as first or second constituents. In fact, the CARIN model (Gagné and Spalding, 2004) ascribes these frequencies a significant role in the activation of thematic relations. If this notion generalizes to effects of grammatical structure, one possible explanation for the different results obtained for coordinative and subordinate compounds could be that the constituents used in subordinate compounds are more consistently involved in subordinate compounds than the constituents used in coordinative compounds are part and parcel of coordinative compounds. According to this notion, the mental lexicon would contain information about the grammatical relationships characters form with other characters in compound words. It is further assumed that a compound word is identified faster, if the relationship is common for a given character. This in turn assumes that the identification of grammatical structure is an integral part of compound word identification. In order to test this notion, future studies should explicitly manipulate constituent-specific structural frequencies separately for coordinative and subordinate compounds.

## General discussion

In the present study, we were interested in finding out whether the grammatical structure of compound words plays a role in compound word recognition in Chinese. This was done by priming the compound word structure to see whether a shared structure between the prime and target will speed up word recognition as measured by lexical decision latency. Two types of structures were tested: subordinate and coordinative structure. The subordinate structure refers to the modifier-head structure where the first constituent (modifier) of a compound word modifies the meaning of the second constituent (head), as in 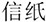, where the first constituent means *letter* and the second *paper*, in referring to *notepaper*. In the coordinative compounds, the two constituent contribute equally to the compound meaning, having either similar or opposite meanings to one another, as in 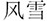, where the first constituent means *wind* and the second *snow*, in referring to *blizzard*.

Experiment 1 was a replication of the methodology of Liu and McBride-Chang (2010) using a more representative sample of Chinese speakers (*n* = 96) than the original study. In addition to priming the compound word structure, also the semantic relationship was varied (related vs. unrelated) between the prime and target. Experiment 1 observed a reliable effect of semantic priming, but in contrast to Liu and McBride-Chang, no evidence for structure priming was obtained. The lack of structure priming is taken to suggest that when the overall compound word meaning is primed, the compound word structure is not active in the reader's mind in the sense that it does not facilitate word recognition.

In Experiment 2, in addition to priming the word structure, the constituents were also lexically primed by repeating the same character either as the first or second constituent across the prime and target compounds. A reliable lexical priming effect was observed for the first and second constituent. The effect emerged both for the coordinative and subordinate compounds. It suggests that constituents play a significant role in compound word recognition in Chinese. In other words, the decomposition route is utilized in the recognition process when accessing the word representation in the reader's mental lexicon. More importantly, a structure priming effect was obtained for subordinate but not for coordinative compounds. Subordinate compounds were recognized faster when preceded by a prime sharing the same structure. This was true when the prime and target shared the same modifier as well as the same head. It suggests that a common lexical element is needed for structure priming to emerge for subordinate compounds. When this result is combined with the absence of structure priming observed in Experiment 1 when the prime and target compounds did not share constituents, it may be concluded that grammatical structure priming is lexically based and that abstract structure priming may not exist.

To sum up the results, the present study demonstrates that processing the grammatical structure can contribute to and be a part of compound word recognition in Chinese. Yet, the structure effect is limited in scope. It was established only for subordinate compounds but it required that a compound word constituent (either the modifier or the head) was simultaneously active with the structure information. On the other hand, when the compound word meaning was primed in the absence of shared constituents between the prime and target, word structure did not facilitate word recognition.

The structure priming effect obtained for subordinate Chinese compounds (see Ji and Gagné, 2007, for analogous findings obtained for relational priming) supports the view that in recognizing two-constituent compounds both constituents need to be accessed to derive the structure and meaning of the word. This makes a lot of sense considering the fact that in Chinese script characters are presented with no spaces between words. Thus, reading Chinese requires readers to consult the adjacent characters in determining whether a character combines with another character to make a compound word or whether it forms an independent meaning. The importance of considering both constituents and their mutual relation is further boosted by the existence of several different types of compound word structures in Chinese. This contrasts with English where the availability of relational information during word recognition has only been found with respect to the modifier (Gagné and Spalding, 2004). In English, compound word structure is mostly limited to the subordinate structure.

The structure effect observed in the present study may be interpreted to support the CARIN (Competition-Among-Relations-In-Nominals) theory put forth by Gagné and Spalding (2004). This theory assumes that combined concepts are formed by binding two constituents with a specific thematic relation; the difficulty of any particular combination is a function of the likelihood of the thematic relation for the particular constituents. Ji and Gagné (2007) extended the theory to the processing of existing compounds in Chinese by demonstrating that thematic relations can be extracted online and utilized in compound word recognition. The present study extends it to also apply to the processing of grammatical structure of Chinese subordinate compound words. The theory further assumes that the difficulty of forming any particular combination is a function of the likelihood of the thematic relation for the particular constituents. Future studies on the role grammatical structure in Chinese compound word identification should examine how the frequency with which particular constituents are involved in different grammatical structures plays out in the recognition process. It is possible that these constituent-specific structural frequencies play a more significant role than the type of grammatical structure itself.

## Ethics statement

All subjects gave written informed consent in accordance with the Declaration of Helsinki.

## Author contributions

LC designed the study, wrote the first draft of the manuscript, took part in the data collection, performed the analyses and contributed to the interpretation of the data. JH contibuted to the study design, wrote the final draft of the manuscript and contributed to the interpretation of the results. FC, JW, WZ and YZ contributed to the compilation of the experimental materials and to the acquisition, analysis and interpretation of the data. They also commented a non-final draft of the manuscript.

### Conflict of interest statement

The authors declare that the research was conducted in the absence of any commercial or financial relationships that could be construed as a potential conflict of interest.

## Footnotes

^1^It is in principle also possible that the pattern of results is due to thematic relations. This explanation requires that the primes of the subordinate compounds share more often the same thematic relation with the target compound than the primes of the coordinative compounds. We did not pay attention to the thematic relations when selecting the stimuli. However, it seems likely that the opposite is true. Coordinative compounds can basically have two thematic relations between the constituents (synonym vs. antonym), whereas the subordinate compounds have more variable relations. If so, the likelihood of the target and prime sharing the same thematic relation would be more probable for coordinative than subordinate compounds. At any rate, future studies should tease apart the effects of grammatical structure and thematic relations.
